# Primary breast lymphoma: a consideration in an HIV patient when a mass is discovered by screening mammography: a case report

**DOI:** 10.1186/1757-1626-1-387

**Published:** 2008-12-11

**Authors:** Olagoke K Akinwande, Robert Paley

**Affiliations:** 1 Department of Radiology, St Agnes Hospital, Baltimore, MD, USA

## Abstract

Primary Breast lymphoma is a rare lesion that has been reported in patients without HIV. However, Primary Breast lymphoma occurring in a patient with HIV has rarely been reported despite the fact that HIV infection is known to increase the propensity to develop certain types of lymphoma. We report a case of an HIV patient with breast lymphoma that was discovered by screening mammography while presenting our argument for more cautionary management in this patient population.

## Background

The breast is a rare site for extranodal lymphomas which tend to occur more commonly in other sites such as the gastrointestinal tract and the central nervous system. HIV (Human Immunodeficiency Virus) infections are known to be associated with extranodal lymphomas, but only a few papers have reported on HIV in association with primary breast lymphoma. Furthermore, none of the cases reported in those articles were discovered on routine breast cancer screening. We report on a case of breast lymphoma in a patient with a history of HIV discovered on routine screening and discuss the argument for more cautionary management in this patient population.

## Case presentation

**Figure 1:**
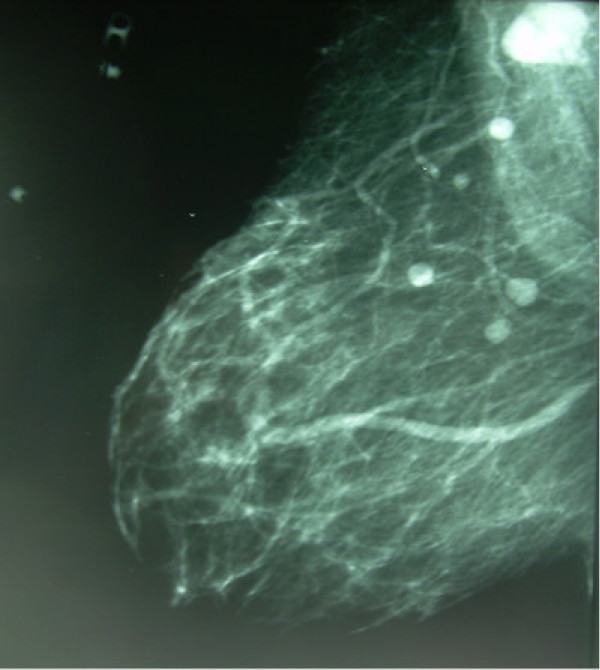
Initial screening mammography showing new multiple circumscribed breast lesions with axillary lymphadenopathy.

**Figure 2:**
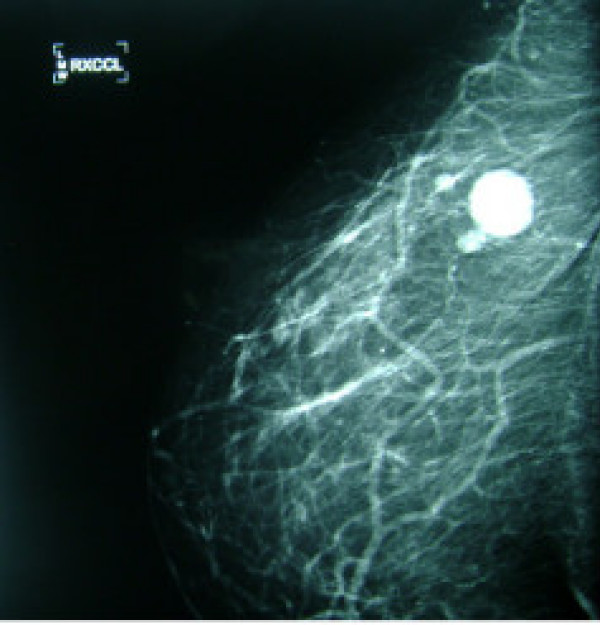
Exaggerated right CC view showing multiple circumscribed nodules in the lateral right breast with the largest nodule measuring about 2 cm.

**Figure 3:**
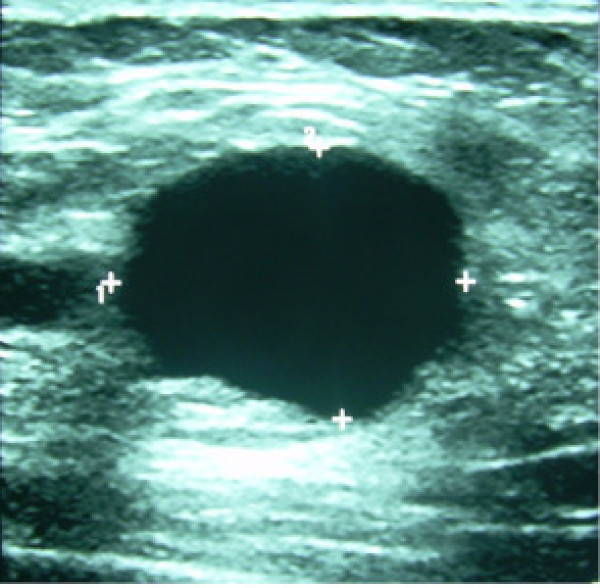
Sonographic evaluation of the right upper quadrant of the right breast showing the dominant lesion as an echo-lucent mass measuring about 2 cm.

**Figure 4:**
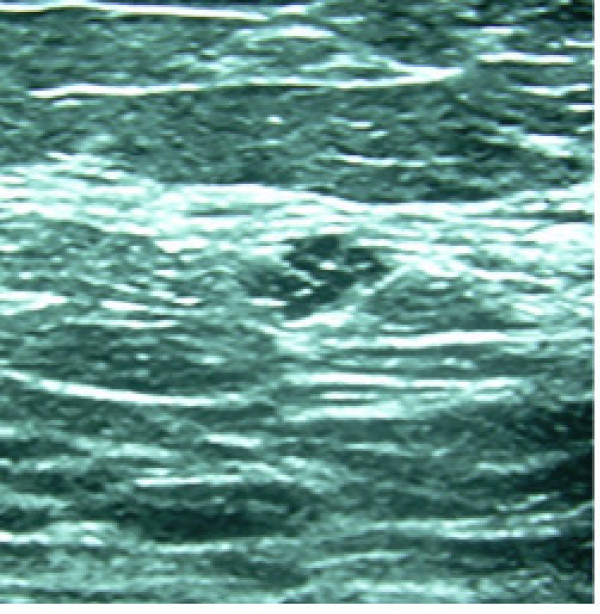
Sonographic image of a group of clustered microcysts found 6 cm from the nipple.

**Figure 5:**
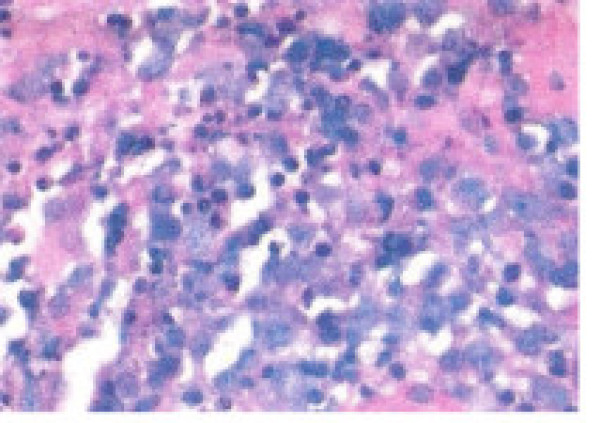
Core biopsy showing infilteration by diffuse large cell lymphoma with necrosis and apoptosis. The cells expressed LCA, CD20, CD10, bcl-6, and Ki-67.

**Figure 6:**
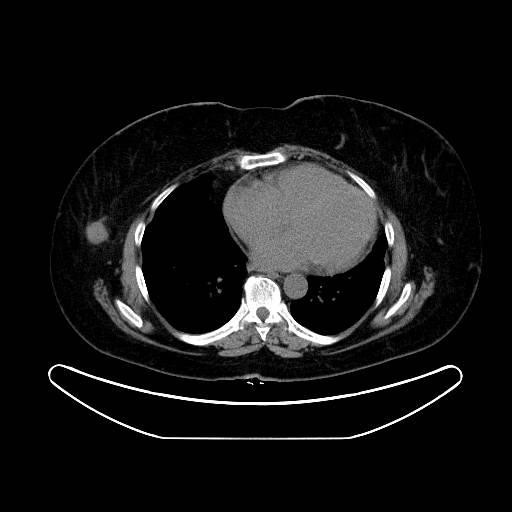
PET/CT scan of the chest showing the mass on the right breast.

**Figure 7:**
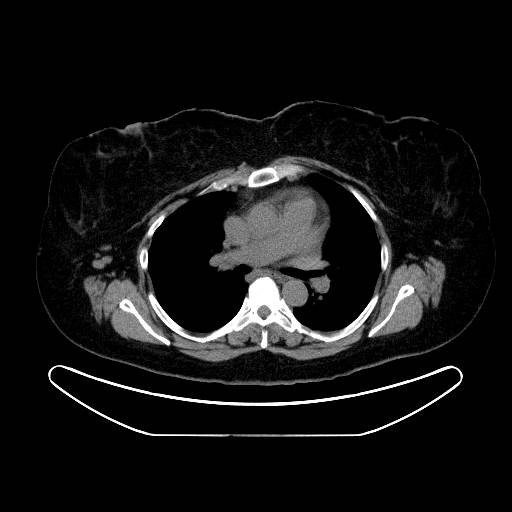
Another slice of the CT scan showing right axillary adenopathy.

Our patient was a 50 year old female with a history of HIV on HAART (Highly Active Antiretroviral Therapy), who came in to our center for routine breast cancer screening. She had a significant family history of breast cancer in her sister, maternal grandmother and her aunt. Her last mammography was two years prior which only showed benign calcifications in both breasts and an enlarged benign-appearing lymph node, hence a BI-RAD 2 (Breast Imaging Reporting Data) designation was given. On the day of presentation, the patient was asymptomatic. She denied any B-symptoms (night sweats, fever, etc) or bone pain. Digital screening mammography was obtained which showed a new circumscribed nodule in the right upper quadrant measuring 2.3 cm, multiple circumscribed benign-appearing lymph nodes in each breast and bilateral axillary lymphadenopathy (fig. 1). A BI-RAD 0 (incomplete) designation was given. Exaggerated right CC view showed multiple circumscribed nodules in the lateral right breast with the largest nodule measuring about 2 cm (fig. 2). Sonographic evaluation was performed on the upper outer aspect of the right breast which showed an echo-lucent mass (fig. 3). Adjacent to that mass was another 9 mm hypoechoic area and an area in the 10 o'clock position consisting of clustered microcysts measuring 6 × 4 × 7 mm (fig. 4). A BI-RAD code of 4 was given for the suspicious lesions and fine-needle cyst aspiration was ordered. During the procedure, core biopsy was initiated because the mass was solid. Pathology results from the breast biopsy showed an aggressive high-grade B-cell lymphoma with positive immunostaining for CD20, CD10 and Bcl-6 (fig. 5). The Ki-67 proliferation index was approximately 80%. PET/CT scan was ordered for staging which showed focal FDG accumulation in the right breast mass with ipsilateral axillary adenopathy (fig. 6 and 7). Right breast lumpectomy with right axillary lymph node biopsy was performed and the patient was started on systemic chemotherapy.

## Discussion

Breast lymphomas have been reported in various publications as a rare cause of a breast mass. Most breast lymphomas are of the Non-Hodgkins type with secondary breast lymphoma being much more common than the primary type. Primary lymphoma of the breast – which is lymphoma confined to the breast and the ipsilateral lymph nodes – accounts for less than 0.7% of all Non-Hodgkins lymphomas (NHL) [1,2]. It has been reported in recent papers that the rare occurrence of extranodal lymphoma of the breast is due to the relative paucity of intramammary lymph nodes [3].

HIV infections are associated with an increased risk of extranodal lymphomas [4]. Furthermore, it has been speculated that the virus affects the breast parenchymal components by decreasing its ability to suppress tumor cells [5]. Despite this, the association between HIV and breast lymphoma while plausible has rarely been reported [6,7]. The establishment of this association relies on larger studies or case series which are not easy to perform due to the rarity of breast lymphoma occurring in conjunction with HIV infection (Chanan-Khan et al. reported only 3 out of the 177 patients in their tumor registry database with NHL concurrently had HIV) [6].

The current literature on breast lymphoma have pointed out certain clues to help in the diagnosis of breast lymphoma that involves both clinical and radiographic features: A breast mass following a current or prior history of diffuse lymphomatous disease, bilateral axillary adenopathy occurring with a breast mass [3,8,9], and a radiographic mass accompanied by other clinical findings such as B-symptoms, bone pain, or a palpable mass. The aforementioned features should arouse suspicion and prompt further workup even if the radiographic mass is benign-appearing or non-specific. Indeed, even though the aforementioned cases might signal the possibility of breast lymphoma there is no pathognomonic radiographic feature to distinguish this disease entity. Mammographic features most commonly seen in cases of breast lymphoma are non-specific: solitary, well or partially defined, non-speculated and non-calcified. On ultrasound, the lesions are most frequently hypoechoic [8-10]. The most helpful characteristic that help signal the need for further workup is the fact that most patients with breast lymphoma are symptomatic. Most patients present with a palpable mass (either focal or diffusely enlarged) and a few report pain. Only a the small subset of these patients are discovered incidently on routine mammography [3,8,10]. The fact that most patients present with a palpable breast lesion is very helpful, but it does not help to distinguish it from the more common breast carcinoma. Differentiation between the two could be made by FNA or core biopsy.

Our patient's lesion fit the criteria for a primary breast lymphoma; there were no extra-mammary sites of involvement other than the ipsilateral lymph-nodes. She did not present with any symptoms at the time of her routine screening mammography which is a rare mode of presentation for this particular lesion. Ultrasonography of the breast lesion showed an echo-lucent mass with round, well defined margins. This lesion is one that is usually suggestive of an incidental cyst but was instead a solid mass which turned out to be lymphoma. Her previous history of HIV should have been a tip off to the possibility of lymphoma. Her viral load and CD 4 levels were at acceptable levels at the time of presentation, but previous non-compliance with her HAART therapy might have resulted in her increased susceptibility to NHL. Her family history of breast cancer and her age were significant risk factors for the more common breast carcinoma which in itself should warrant cautionary workup even in light of her benign-appearing radiographic findings.

From the discussion above, we speculate that HIV which increases the risk of extra nodal lymphomas might also increase the risk of lymphomatous breast involvement. However, while most cases of breast lymphoma were found in patients without HIV, more studies are need to ascertain if there is an increased rate of breast lymphomas in patients with HIV compared to patients without.

## Conclusion

Breast lymphomas can be discovered on routine screening, therefore it should always be included in the differential diagnosis of an incidental breast mass. We also propose that since HIV patients are at increased risk for extranodal lymphomas, extra caution must be provided for patients with masses on mammography.

## Consent

Written informed consent was obtained from the patient for publication of this case report. A copy of the written consent is available for review by the Editor-in-Chief of this journal.

## Competing interests

The authors declare that they have no competing interests.

## Authors' contributions

OA conceived the study, participated in acquisition of the data, compilation of relevant literature and drafted in the preliminary and final manuscript. RP was involved with the patient's care, and was involved in the provisional and final diagnosis. He also reviewed the manuscript.
